# Speech Interaction to Control a Hands-Free Delivery Robot for High-Risk Health Care Scenarios

**DOI:** 10.3389/frobt.2021.612750

**Published:** 2021-04-08

**Authors:** Lukas Grasse, Sylvain J. Boutros, Matthew S. Tata

**Affiliations:** ^1^ Canadian Centre for Behavioural Neuroscience, Department of Neuroscience, University of Lethbridge, Lethbridge, AB, Canada

**Keywords:** speech recognition, assistive robotics, COVID-19, medical robotics, human-robot interaction

## Abstract

The Covid-19 pandemic has had a widespread effect across the globe. The major effect on health-care workers and the vulnerable populations they serve has been of particular concern. Near-complete lockdown has been a common strategy to reduce the spread of the pandemic in environments such as live-in care facilities. Robotics is a promising area of research that can assist in reducing the spread of covid-19, while also preventing the need for complete physical isolation. The research presented in this paper demonstrates a speech-controlled, self-sanitizing robot that enables the delivery of items from a visitor to a resident of a care facility. The system is automated to reduce the burden on facility staff, and it is controlled entirely through hands-free audio interaction in order to reduce transmission of the virus. We demonstrate an end-to-end delivery test, and an in-depth evaluation of the speech interface. We also recorded a speech dataset with two conditions: the talker wearing a face mask and the talker not wearing a face mask. We then used this dataset to evaluate the speech recognition system. This enabled us to test the effect of face masks on speech recognition interfaces in the context of autonomous systems.

## Introduction

On March 11th, 2020, the World Health Organization (WHO) declared Covid-19 to be a global pandemic (Huang et al., 2020), however the transmission and impact of the virus has varied tremendously across regional, racial, and socioeconomic boundaries. Of particular importance and concern is the role of front-line health care workers in spreading the virus (Casini et al., 2019) and the extra burden placed on those workers in situations of high transmission risk. For example in China, a study that surveyed health care workers in hospitals found that half of the employees were depressed (50.7%), close to half of them had anxiety (44.7%), over a third of them suffered from insomnia (36.1%) (Li et al., 2020), and a little under three quarter of them were facing psychological distress (Tomlin et al., 2020).

This burden faced by health-care workers is compounded when those workers are responsible for the mental and physical health of aging patients. Although the Covid-19 pandemic has affected people all over the globe, it has had a disproportionately strong effect on the aging population and their care givers. For example, as of September 2020 there have been just over 146,000 cases in Canada. Of these, 10,549 cases were staff at long-term care facilities and 18,940 were residents of such facilities. Since senior citizens account for 77% of the deaths related to SARS-CoV-2 (NIA, 2020), there was an immediate need early in the pandemic to reduce the rate of transmission to people who live in care facilities for the elderly and the care-givers who work with them. One common strategy has been near-complete lockdown of such facilities. Although effective at reducing the risk of transmission into the resident population, this approach has the unwanted consequence of isolating residents from loved ones at a profoundly stressful time. The longer-term consequences of this physical and social isolation on the mental wellness of the aging population is not yet known. Here we describe an end-to-end robotics solution to break the physical isolation of lockdown in long-term care and similar facilities.

Robotics is a promising area of research that can contribute to an effective response to pandemic across a variety of health care scenarios. Uses have been proposed and developed ranging from assistance during Ebola outbreaks (Yang et al., 2020) to supporting children during their stay in a hospital (Joseph et al., 2018). Robotics and other autonomous systems offer the distinct advantage of uncoupling physical interactions between people by providing the option of interaction-at-a-distance. This enables robots to act as a physical link between people who cannot come into close contact. Widespread use of such systems could act as a surrogate in place of real physical interaction during periods of high risk of disease transmission.

One barrier to adoption of robotics in health care environments is the human-robot interaction (HRI) component. The research performed in this paper uses speech as a modality for interaction, in order to lower the learning curve for end users interacting with robots. Speech is an intuitive and powerful means of interaction between humans and robots, and speech recognition is increasingly being adopted for HRI in humanoid robotics (Stiefelhagen et al., 2004; Higy et al., 2018; Kennedy et al., 2017). However, speech still remains underexplored in industrial collaborative robotics. A goal of this paper was to provide insight into the scientific and technical challenges of audio HRI in complex collaborative robotics.

According the World Health Organization, the coronavirus that causes COVID-19 is transmitted by various modes, but mainly during one of two classes of interactions between individuals: either close contact that allows direct exposure to respiratory droplets, or contact with contaminated surfaces enabling the virus to be transported to the nose or mouth by the hands. Robots cannot contract respiratory diseases and do not cough or sneeze, so using robotic systems as a physical link between individuals breaks the direct respiratory transmission mode. However, most robotic systems employ at least some degree of hands-on operation so that human users can provide instructions to the robot (e.g., via a keyboard or tablet computer). This interaction exposes the risk of transmission via contaminated surfaces. A hands-free solution is needed. Here we present an end-to-end system for robotic delivery of items from a visitor to a resident of a care facility. The proposed system can operate without supervision by a facility worker thus reducing their workload and diminishing their exposure and spread of the virus. Importantly, it is controlled entirely by audio interaction for hands-free use so that both direct respiratory and indirect surface transmission modes are broken.

One novel contribution this paper makes is the evaluation of the effect masks and accented speech have on speech recognition interfaces for robotics in real-world environments. The demonstration of an end-to-end self-sanitizing delivery system is also a unique demonstration that can provide a useful starting point for roboticists looking to build such systems for real world environments. Our system demonstrates how speech control can be integrated into a robotics project, enabling users to directly communicate with robots naturally. This paper has two intended audiences. The first is speech recognition researchers who are curious about the distortion effects of masks, and will find our analysis of speech recognition performance under different mask conditions to be informative. Secondly, roboticists who are trying to develop automated delivery systems will find the technical implementation of the end-to-end solution to be one path to solve the typical problems that arise in this scenario.

## Materials and Methods

The goal of this research was to improve the quality of life and reduce the isolation of residents in facilities with a high risk of disease transmission during the pandemic. We sought to develop a system that can deliver items from visitors to residents using end-to-end voice interaction to prevent physical contact with surfaces. The robot makes use of a custom speech recognition interface to interact with humans at a distance, thus reducing the transmission of pathogens. This section begins by outlining the robotics hardware platform on which the system was demonstrated, then delves into the speech recognition interface used to control the robot, and finally outlines the implementation of a human-robot interaction workflow using a state machine. All custom software was developed using the *Python* programming language.

### 0.1 Robotics Platform

This section outlines the robotics platform and other hardware used to demonstrate the system in this paper. The robotics platform used was a Turtlebot 2 mobile base consisting of a Kobuki base with proximity sensors, and an Orbbec Astra Pro Depth Camera for mapping, navigation, and obstacle avoidance. The Turtlebot 2 uses an Acer netbook to run the mapping, navigation, and other aspects of the mobile base. We mounted a Raspberry Pi 3 Model B to the base of the turtlebot and attached microphones from a Logitech C920 webcam to the top of the robot. This raspberry pi was used to run the speech recognition interface described below. All communication between components of the Turtlebot 2 as well as the speech recognition system on the Raspberry Pi ran through Robot Operating System (ROS) modules. Importantly, by using ROS as a middle layer, the system is scalable to larger ROS-based rover platforms in the case that the Turtlebot 2 is insufficient for a particular use case.

### 0.2 Depth Camera

The depth camera was the Orbbec Astra with a depth image size of 640 × 480 (VGA) 16 bit @ 30 FPS. It has a scanning range from 0.4 to 8 m. The field of view consists of 60°horizontally, 49.5°vertically and 73°diagonally. It also has an infrared and RGB sensor (Astra, 2020).

### 0.3 Mapping and Navigation

The mapping was created using Robot Operating System 3D Robot Visualizer (RVIZ) (Dave Hershberger and Gossow, 2020) and gmapping (Gerkey, 2020a) packages. The gmapping package provided the turtlebot with the laser-based simultaneous localization and mapping (SLAM) node using the depthimage_to_laserscan package. Since it was impossible during the 2020 pandemic to work within the setting of a care facility, we demonstrated our system in a typical academic research building. To control which rooms we wanted to map, we used the turtlebot_teleop package, created by Wise (2020), which provided us with manual control of teleoperation using a keyboard. During this pre-mapping phase, we achieved a better map by occasionally stopping and slowly rotating the robot to draw an accurate representation of the objects and obstacles around it. We used the publish point feature in RVIZ and manually integrated the waypoints in a python dictionary to obtain the coordinates of the rooms that we wanted to include in the turtlebot’s database. Each waypoint consisted of five different entries: the exact coordinates, and four nearest neighbours. The nearest neighbours were intended to be used as a fallback option. In the event that the turtlebot could not properly plan a trajectory to one set of coordinates, it fell back to the next set until a proper plan was made available to be followed. The map was stored using the map_server package, created by Brian Gerkey (2020), this produced two files (.pgm and .yaml) that were used later with the AMCL package. The AMCL package created by Gerkey (2020b) was used with the previously generated map to allow the turtlebot to navigate to specified waypoints provided by the audio interaction interface.

### 0.4 Sanitization Pod

Another technology that has shown promise for reducing virus transmission rates is Ultraviolet (UV) light sanitization. A recent study on UV light has shown it to be effective on killing Covid-19 virus (Kitagawa et al., 2020) in their study, the authors have demonstrated that a 222 nm Ultraviolet C (UV-C) irradiation for 30 s resulted in 99.7% decrease of SARS-CoV-2 virus. The combination of UV light sanitization and robotics is a powerful combination for fighting the war against Covid-19. An example of this combination is a robot developed by MIT and Ava Robotics that can sanitize warehouses through the use of UV-C light (Gordon, 2020). Robots can assist health care employees with trivial tasks that reduce human exposure to and the spreading of the SARS-CoV-2 pathogen.

Our system as conceived in this context is not fool-proof, for example if the robot encountered an infectious individual while navigating through the space, it is possible that it could transmit pathogens. Since many transmissible pathogens can live on surfaces for minutes to hours, we included an intermediate behaviour for the robot in which it brings the item to be delivered to a location where it can be cleaned. For our demonstration, we built a custom enclosure with an opening to represent a station for either automatic or manual sanitization of the to-be-delivered item. We envision a more elaborate future implementation that might involve automatic UV-C or similar systems.

An important aspect of voice communication is acknowledgement that the receiver is indeed listening to the speaker’s instructions. Humans use behaviours such as head-turning and sometimes subtle facial gestures to convey attentiveness. To provide acknowledgement of voice commands, we used a voice-activity detection algorithm (provided by WebRTC) with an LED indicator to show that the robot was triggered to be in listening mode.

### 0.5 Speech Recognition Interface

The speech recognition interface consisted of multiple components that record and understand the speech of the person using the delivery robot. The following section gives a high-level overview of the speech recognition interface. The process started with voice activity detection (VAD) and speech recognition. We compared two commonly used open source speech recognition systems in our research, Mozilla DeepSpeech and Kaldi. Each speech recognition system used a custom language model with a vocabulary that was restricted to the specifics of the delivery task. Once a sentence was recognized the user intent was parsed from the sentence using simple rules. Next, we consider the specifics of each component of the system.

#### 0.5.1 Speech Recognition and Custom Language Model

The first speech recognition system was implemented using WebRTC (Google, 2020b) for voice activity detection (VAD) and used the DeepSpeech architecture demonstrated by Hannun et al. (2014) for speech recognition. Specifically, we used the implementation from Mozilla (2020). This implementation contains a model that runs using Tensorflow Lite (Google, 2020a). This allowed us to run the speech recognition system on a raspberry pi in real-time. The system started by performing VAD on each audio frame using WebRTC and then added each incoming audio frame to a ring buffer. If the ratio of frames containing speech exceeded a threshold, the existing frames from the buffer were fed to the speech recognition system. New frames were continuously fed to both the speech recognition system and the ring buffer until the ratio fell back below the threshold. Some adjustment of the voice activity threshold were required, but once the correct threshold was determined the system was quite effective at identifying the onset of a voice.

Mozilla Deepspeech uses KenLM (Heafield, 2011) as the language model used during decoding the speech from the neural network. In this research we trained a custom KenLM language model that was used to recognize specific sentences related to initiating the robot to deliver a package, confirmation of a correct delivery location, confirmation of receipt of a package, etc. Using a reduced custom model substantially increases the accuracy of the speech recognition system’s performance in real-world environments. The custom language model was a 3°g KenLM model trained with example sentences. During inference we set the language model alpha value to 0.931,289 and the beta value to 1.183,414 as these were the default values used in DeepSpeech.

The second speech recognition system we used was Kaldi (Povey et al., 2011), and the Vosk API (Cephei, 2020). For this system we used the built in voice activity detection and the *vosk-model-small-en-us* model, which was lightweight and can run on small single board computers. This model also uses the Kaldi Active Grammar feature, which enabled us to dynamically change the vocabulary of the model to only include words relevant to the delivery task. We used the same set of sentences and words used to train the previously described custom language model for DeepSpeech.

#### 0.5.2 Intent Parsing

The next step in the speech recognition system was parsing the user’s intent from the recognized speech. This was greatly simplified due to the use of the restricted language model described previously. The main type of intent parsing that occurred was detecting when a user intended to initiate a delivery, and then parsing out the location of the delivery. The first step in achieving this was to ensure a recognized sentence started with the word “robot”, which implied that the user was addressing the robot. Once a sentence that started with the word robot had been recognized, the next step was parsing the location from the string. The language model was restricted such that all the delivery sentences contained the phrase “deliver this package to” as part of the sentence. An example of this is the sentence “hey robot please deliver this package to room A”. The fixed structure of these sentences enabled us to split the recognized string on the words “package to” and take the remaining part of the string as the selected room for delivery.

Another type of intent parsing that occurred in our speech interface was to obtain confirmation from a user: either confirmation of a correct intended delivery location, or confirmation of successful package delivery. The first confirmation happened after the selected room identifier was parsed out of the recognized sentence. The robot used the Text-To-Speech system described in the next section to confirm with the user as to whether the room was correctly understood. The user then confirmed the location as correct or rejected the location. To do this, the language model contained multiple sentences containing various confirming statements such as “yes”, “yes that is correct” etc. and other rejection statements such as “no”, “no that’s wrong”, etc. The confirmation intent parsing step then performed keyword spotting over the recognized string to see if any of the rejection statements were present. If they were present the robot rejected the selected location and informed the user. If the sentence contained confirmation statements such as “yes” the robot then performed the delivery. The final case is one in which the perceived sentence did not contain any of the keywords, in which case the system continued listening for a confirmation or rejection.

#### 0.5.3 Text-To-Speech

The system included a Text-To-Speech (TTS) functionality that enables it to speak to users. This is an important component of the Human-Robot Interaction as it is the primary method through which the robot communicates after parsing the delivery location and during delivery confirmation. The TTS system used was the Ubuntu say command, which uses the GNUstep speech engine created by Hill (2008). The TTS system operated using a ROS python script; when a string was published to a TTS topic the TTS system executed the say command using the subprocess library. This enabled the TTS system to be easily triggered via ROS from any device connected to roscore over the network.

### 0.6 State Machine for Human-Robot Interaction

**FIGURE 1:**
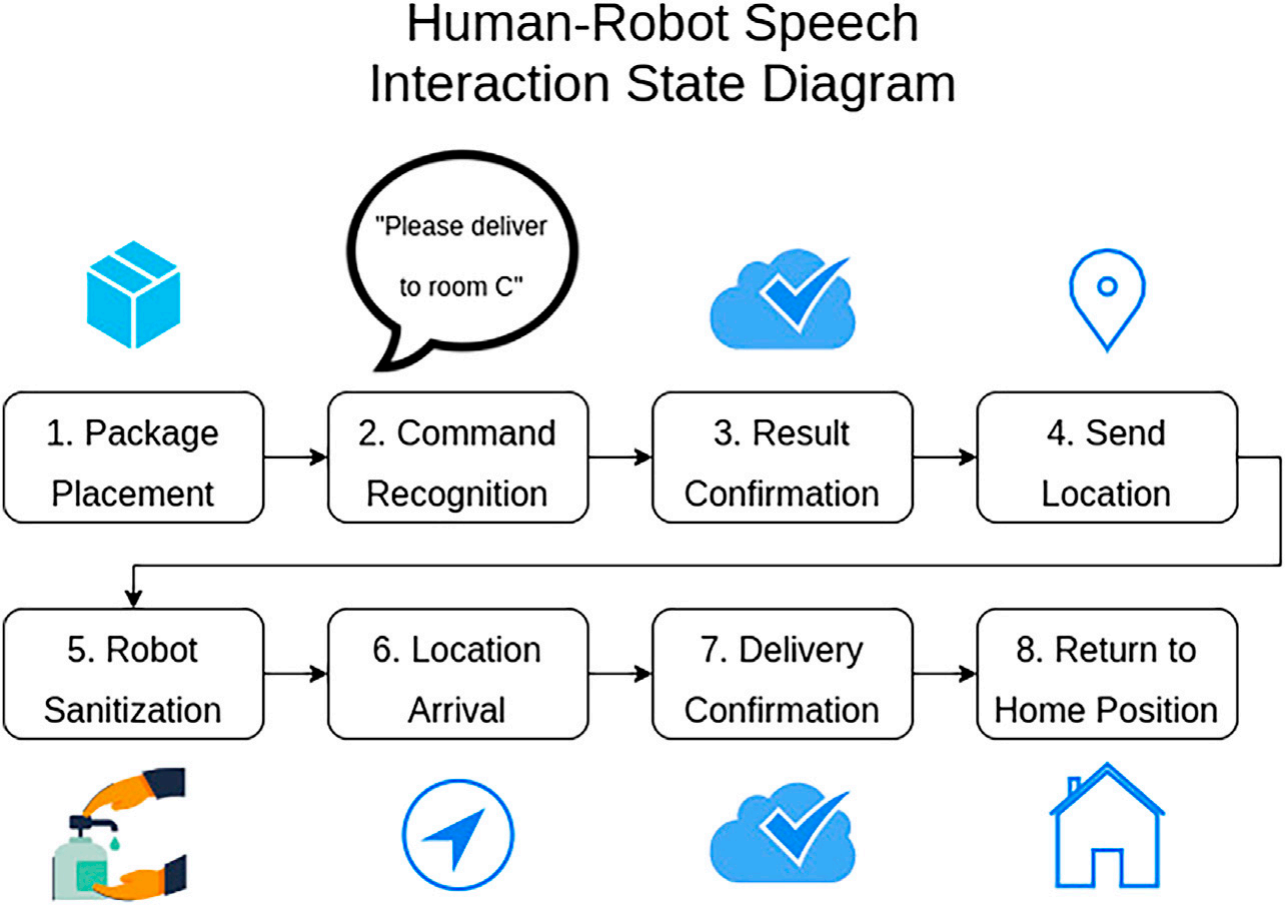
The Human-Robot Interaction Workflow. Delivery was triggered and confirmed by speech interaction between a visitor and the robot. The robot navigated to the delivery target waypoint, with a stop to clean the item with UV-C light. At the target waypoint the robot used speech to confirm delivery and then returned to its home position. Note that the entire interaction required no physical interaction with surfaces on the robot and, except for placing and retrieving the item in the basket, could be conducted from a distance of as much as several meters away.

All of the components demonstrated so far were connected together into a complete system using a state machine that communicated over ROS and coordinated the various aspects of the human-robot interaction. The state machine and interaction process are outlined in Figure 1.

The state machine facilitated an interaction scenario in which a visitor to a long-term care facility wishes to deliver a package to an at-risk individual that resides in the facility. The visitor initiated the interaction with the robot by placing a package in the robot’s delivery basket and then speaking to the robot. Then the visitor instructs the robot on which room to deliver the package to by saying a sentence such as “Hey robot, please deliver this package to room E3”. Once the robot has successfully parsed a delivery sentence the state machine advances to the confirmation state. In this state the robot repeats the room number to the human and asks if the room number is correct.

Once the robot received verbal confirmation, the state machine proceeded to the next state, which sent the room id over ROS to the navigation system. The navigation system then mapped the name to coordinates using the turtlebot’s database. Next, the robot sanitized itself by navigating into the UV-c sanitization pod. Once the robot had waited for the correct amount of time in the pod it navigated to the coordinates for the target room. Arrival at the delivery target waypoint triggered the delivery confirmation state. In this state the robot used the TTS system to inform the recipient that they have a package and should remove it from the basket. After a time delay to account for the removal of the package the robot asked for confirmation that the package has been removed. Once the robot received this verbal confirmation, the state machine entered the final state, in which the robot navigated back to the home position.

The state machine included an error state. The error state communicated to the user that the robot did not understand a command and then returned to the initial command recognition state. This error state was specifically used during the command recognition and confirmation stages to provide a way for the robot to reset itself if instructions were unclear.

### Methodology

Our tests of the implemented system consisted of a test of the delivery scenario from start to finish, and an in-depth test of the speech recognition component. The setups of these experiments are described below.

#### 0.6.1 Delivery Scenario Evaluation

**FIGURE 2:**
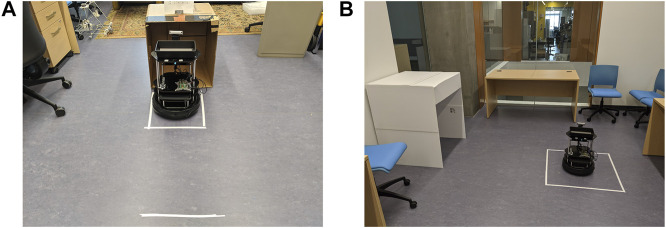
Experimental setup for speech recognition evaluation and delivery scenario evaluation.

We verified the system’s functionality by completing an end-to-end speech-controlled delivery using all of the components described in the implementation section. We envisioned a scenario in which a visitor to a care home would want a small item such as a note or gift to be delivered to a resident in a lock-down situation. Thus the test ran between two labs at the Canadian Centre for Behavioural Neuroscience at the University of Lethbridge. One lab was designated as the start point where the visitor would give the item to the robot and provide voice instructions about where to deliver it. This space also contained a designated parking area for the robot and a box intended to simulate the sanitization pod. This lab was labelled lab c (see Figure 2). The destination for the delivery was set to a second lab that was in the same building and connected by a hallway to the first lab. This lab was labelled lab t. The hallway contained garbage/recycling bins which provided a realistic real-world environment for navigation. Both rooms were on the same floor, as our robotics platform cannot navigate between floors. This is a critical challenge that would be important for future research in developing delivery systems.

The speech recognition system used in the test had a language model that recognised sentences such as *robot please deliver this package to room c* or *robot take this package to room t*, where *room c* and *room t* were the only valid room names. During the test a light package was placed in the delivery basket in lab c and removed in lab t upon successful delivery.

#### 0.6.2 Speech Recognition Evaluation

To evaluate the performance of the speech recognition system in real-world conditions, we recorded audio from 13 participants asking the robot to deliver a package to a room they selected from a list of 30 possible rooms. Audio was recorded in a typical office space with background heating/ventilation as the main source of noise (the RT60 of the room was 0.33 @ 1,000 Hz and the average SNR was 10.92 dB). Each possible room was a randomly generated combination of a single letter and single numerical digit, e.g., E4. The audio was recorded using microphones mounted on the turtlebot. Since viruses cannot be transmitted to a robot, the visitor need not maintain any particular distance from the robot. Thus, participants stood facing the turtlebot behind a line marked on the ground 61 cm back from the robot’s position to speak the commands. The recording setup is shown in Figure 2.

A hallmark of the COVID-19 pandemic was the widespread requirement to wear a face mask in public spaces. Since such masks are known to impart a low-pass filter to speech (Corey et al., 2020), we considered whether our speech recognition system would be negatively affected if users were wearing masks. We therefore recorded speech using two conditions, one with the participant wearing a face mask and another with the face mask removed. This enabled us to calculate whether or not a face mask would impede the accuracy of each speech recognition system. For each condition we recorded five trials for a total of 10 trials per participant. Each participant wore their own personal mask. The masks consisted of a variety of cloth, disposable polyester, and other masks.

## Results

### 0.7 Experiment 1: Delivery Scenario Evaluation

**FIGURE 3:**
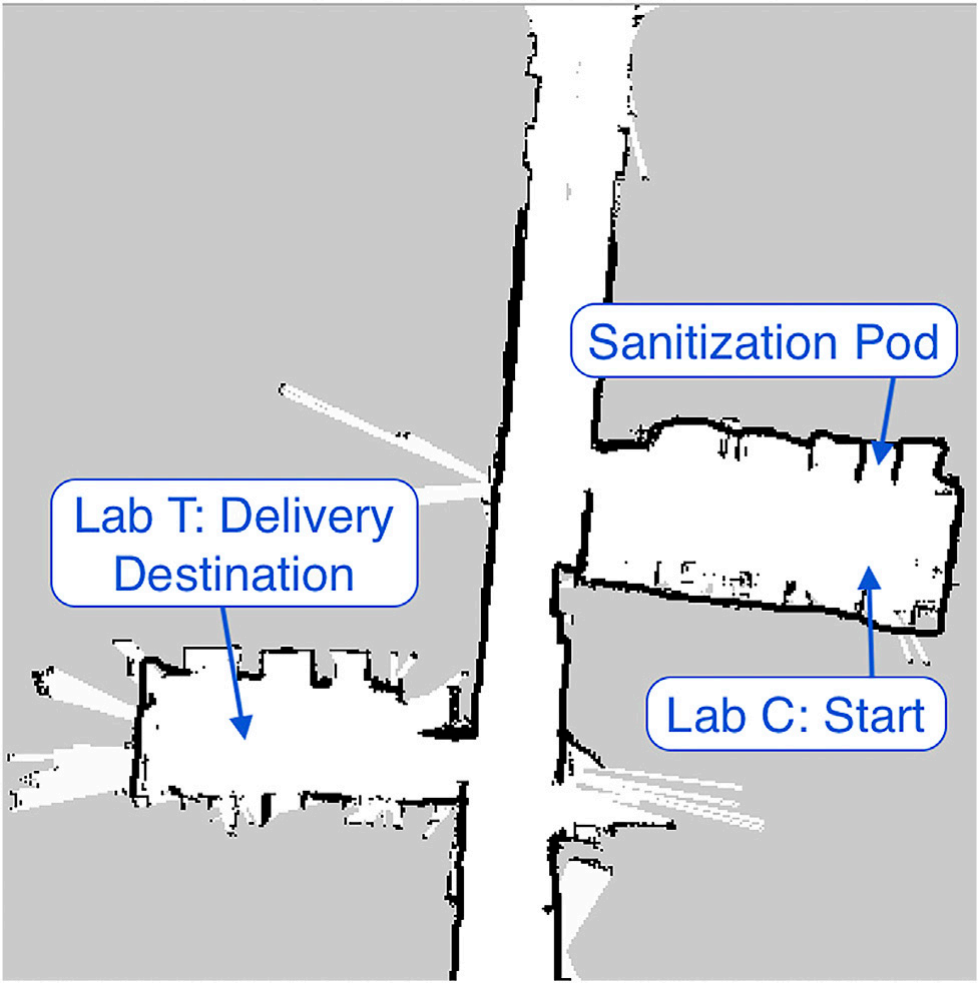
Map generated for turtlebot navigation using the ROS gmapping package and an astra depth camera.

The first evaluation we performed of the delivery robot was an end-to-end test delivery of a package from a starting location to an end location, as outlined in Section 0.6.1. The first step in this test was the manual generation of a map using the ROS gmapping package. The turtlebot was manually navigated using the teleop package and the generated map was saved for use during navigation. The map is shown in Figure 3. The coordinates of the starting point, sanitization pod, and delivery destination, were then recorded and added to the python dictionary described in Section 0.3. The command recognition component of the state machine was configured such that the recognition of the phrase *Room T* triggered a delivery to lab T and *Room C* triggered a delivery to lab C. The previously described DeepSpeech system was used for this step. The coordinates of the starting point in lab C were also used as the location of the home position.

A package was placed in the robot’s delivery basket. After receiving a verbal delivery command, the robot successfully recognized and parsed the sentence asking it to deliver the package to room T. The robot then navigated into the sanitization box, waited for the correct amount of time, and then autonomously navigated to lab T. Once the package was removed and the robot received verbal confirmation it returned to the initial starting point in lab C. A video demonstration is available at^1^.

### 0.8 Experiment 2: Evaluation of Delivery Location Recognition System

The use of audio interaction to achieve hands-free autonomy for the delivery robot was a key goal of this research. We explored factors related to the success of potential failure of each speech interaction system. We were particularly interested in two factors that might influence the usefulness of audio interaction in this use case: the use of a protective face mask, and the challenge of recognizing the speech of users whose first language is not English. Specifically, we evaluated each system’s delivery location recognition component as described in Section 0.6.2. Thirteen participants contributed five audio samples for each experimental condition: with and without a protective cloth mask covering the mouth and nose. The audio was recorded using the microphones on the robot. We recorded audio from both native English speakers and non-native speakers.

**FIGURE 4:**
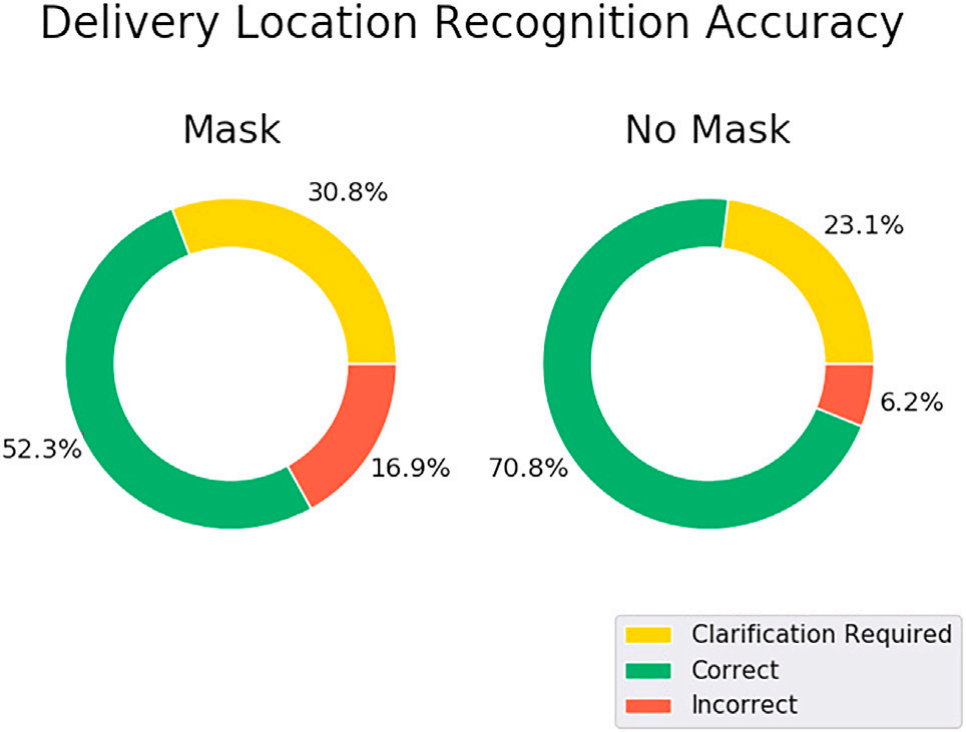
First-pass outcomes of each recognition system with **(left)** and without **(right)** a face mask. Recognition was scored as *correct* when the intended target room was successfully recognized the first try. Recognition was scored as *clarification required* when the state machine needed to enter the error state and ask the user to repeat the instruction. Recognition was scored as *incorrect* when the system recognized the wrong room. This mode would require the user to reject the recognized room during the confirmation step and repeat the instruction.

We measured accuracy to choose the correct target room by running each speech recognition system on the pre-recorded audio and parsing the results using the same approach as the command recognition step of the state machine. Accuracy was then calculated across all trials for each condition as the percentage of destinations that were parsed and determined successfully. The results of the experiment are shown in Figure 4. The mask-off condition outperformed the mask-on condition for both systems, correctly recognizing **70.8** and **73.8%** of the destinations as opposed to **52.3** and **60.0%** for the mask-on case for the DeepSpeech and Kaldi/Vosk systems respectively. We also compared the results between participants who learned English as a first language and non-native speakers of English. The results are shown in Table 1. We found that the speech recognition system performed poorly with non-native English, particularly when the speaker was wearing a mask.

**TABLE 1:** First-pass Recognition Accuracy compared between participants who learned English as their first language vs. English as a second language. The best performance is highlighted in bold. Accuracy is averaged over all five recordings for each condition from all 13 participants.

Delivery Location Recognition Accuracy %				
Mask Condition	No Mask		Mask	
Approach	DeepSpeech	Kaldi/Vosk	DeepSpeech	Kaldi/Vosk
English is First language	**75.0**	**75.0**	62.5	70.0
English is second language	64.0	72.0	36.0	44.0

## Discussion

The Covid-19 pandemic has put an enormous strain on front-line medical workers and threatened the lives of millions worldwide. The widespread effects of the virus have also disproportionately effected our aging population, who account for 77% of Covid-19 related deaths. The social and mental health impacts of lockdown measures in long-term and assisted-living care facilities for seniors, even for those who never experience the disease, are not yet known but are likely to be severe. We sought to develop an end-to-end autonomous delivery system that could break the physical isolation of care-home residents by delivering physical items (such as gifts, letters, etc.) from visitors to residents. The system needed to meet two criteria: 1) break the direct respiratory transmission pathway by using a mobile rover platform and 2)break the surface transmission pathway by providing an end-to-end hands-free speech control system. The system needed to be intuitive to use and good at trapping errors.

We built a successful system based on a ROS-controlled Turtlebot2 mobile base and free-field microphones. In ideal conditions the system demonstrated good first-pass accuracy (75% for native English speakers without masks) at understanding the target destination for the delivery. In the first-pass failure mode, 21% of outcomes were cases of the robot failing to understand any target instruction and, thus, asking for the instruction to be repeated. Only 6% of first-pass interactions resulted in the robot understanding the wrong target destination. Coupled with simple error-trapping in the confirmation step, we believe the system could perform quite well under ideal conditions.

One goal of this work was to compare commonly used speech recognition packages (DeepSpeech vs. Kaldi/VOSK). Whereas the two approaches performed identically given the hypothetically ideal case of native English speakers without masks, we found that Kaldi/VOSK handled the less-ideal case of users wearing masks. In that case Kaldi/VOSK showed a lower tendency to have high confidence in the wrong room (16.9% for DeepSpeech vs. 1.5% for Kaldi/VOSK).

There are, however, a number of challenges that were uncovered by this research. First, it is evident that face masks cause problems for automatic speech recognition. This is unsurprising given that they effectively low-pass filter the acoustic signal (Corey et al., 2020). One solution might involve training a custom acoustic model for the speech recognition system trained on audio that is recorded from people wearing masks. Alternatively, an existing speech dataset could be modified to simulate the acoustic effect of a mask. A second important challenge is that speech recognition systems may perform worse for non-native speakers of English. This is a known problem of speech recognition systems in general trained with speech from native English speakers (Hou et al., 2019; Derwing et al., 2000). Importantly though, the problem of recognizing non-native English speech and the problem of recognizing speech with a face mask seem to be interactive (such that only 36% for DeepSpeech and 44% for Kaldi/Vosk of first-pass recognitions were successful in this worst-case scenario). Creation of a more robust acoustic model could increase the reliability and widespread usefulness of the system.

Here we demonstrated the usefulness and some of the challenges associated with hands-free audio control of robotics. By building the system around a set of ROS modules, the system is both scalable and portable to other robot systems that make use of the ROS platform. Although this research is described entirely within the context of autonomous delivery in a health-care isolation scenario, it is easy to imagine related use cases in which hands-free control of a mobile robot platform might be advantageous. It is our hope that the present study draws attention to the important contribution that audio AI can make to sound-aware robots across a wide-range of use cases.

## Data Availability

The datasets for this study have not been published, as they contain personally identifiable human data. They can be made available on request.
